# Hypoplastic lower extremity in a child with a mosaic PIK3CA variant

**DOI:** 10.1016/j.jdcr.2026.03.043

**Published:** 2026-03-30

**Authors:** Sabine Obagi, Joseph Dodson, Timothy James Maarup, Jingyun Gao

**Affiliations:** a College of Medicine, University of Arizona, Tucson, Arizona; b Department of Dermatology, Kaiser Permanente Los Angeles Medical Center, Los Angeles, California; c Department of Genetics, Kaiser Permanente Los Angeles Medical Center, Los Angeles, California

**Keywords:** growth disorders, mosaicism, pediatrics, *PIK3CA*, PIK3CA-related altered growth spectrum, vascular malformations

## Introduction

Activating somatic variants in *PIK3CA* produce a wide clinical spectrum of segmental vascular and growth abnormalities traditionally grouped under the PIK3CA-related overgrowth spectrum (PROS).^1^

However, recent literature has demonstrated that these disorders extend beyond isolated overgrowth. In particular, undergrowth or hypoplastic phenotypes associated with activating *PIK3CA* variants have led authors to propose the broader term PIK3CA-related altered growth spectrum (PRAGS), which encompasses both hypertrophic and hypoplastic manifestations.^2^

The *PIK3CA* gene encodes the p110α catalytic subunit of class I phosphoinositide 3-kinase, regulating cellular proliferation and growth through the PI3K-AKT-mTOR pathway.^3^^,^^4^ While PROS phenotypes commonly feature segmental hypertrophy, capillary-venous malformations, and adipose or skeletal overgrowth,^5^ hypoplastic phenotypes remain underrecognized and may complicate diagnosis.

We present a pediatric case of unilateral lower-extremity undergrowth associated with a mosaic *PIK3CA* p.Met1043Ile variant, contributing to the growing recognition of hypoplastic presentations within PRAGS and illustrating diagnostic pitfalls when early assessments focus primarily on overgrowth.

## Case report

A 6-year-old male with a genetically confirmed PIK3CA-related growth disorder presented to our vascular birthmark clinic for evaluation of right lower extremity asymmetry.

### Early history

At age 3, the patient was evaluated by an outside orthopedic provider for presumed left leg hemihypertrophy in the setting of a right-sided capillary malformation. Early imaging documented congenital peripheral arteriovenous and capillary-venous malformations involving the right buttock and extending to the right foot. No abnormalities were identified in the contralateral limb. His medical history included developmental delay and elevated body mass index (23.6 kg/m^2^). Head circumference values were not available in the documentation reviewed.

### Genetic testing and initial workup

A lesional biopsy from the right lower extremity underwent targeted next-generation sequencing as part of a somatic overgrowth panel (University of Pennsylvania) and identified a mosaic activating *PIK3CA* variant (c.3129G>A; p.Met1043Ile) with a variant low allele fraction of approximately 5.0% to 6.9%, consistent with mosaicism. Sequencing was performed on lesional tissue obtained from the right lateral calf at the site of a capillary-venous malformation. No biopsy of the left leg was performed, as there were no cutaneous or vascular malformations warranting sampling.

### Treatment course

In June 2023, alpelisib (Vijoice, Novartis, Basel, Switzerland), a PIK3⍺ inhibitor, was begun at a prior institution. At the time, the limb asymmetry was interpreted as left-sided hemihypertrophy. The patient underwent a brief trial, discontinued at age 5. At the providing institution, follow-up was limited despite multiple outreach attempts. The family reported increased appetite and behavioral changes during therapy, and medication administration was shared among several caregivers, making dosing consistency difficult to assess. The patient’s mother occasionally paused doses in response to these symptoms. During this period, care for his vascular and growth concerns was fragmented across 3 institutions, and insurance difficulties contributed to interruptions in continuity.

### Current presentation

**Fig 1:**
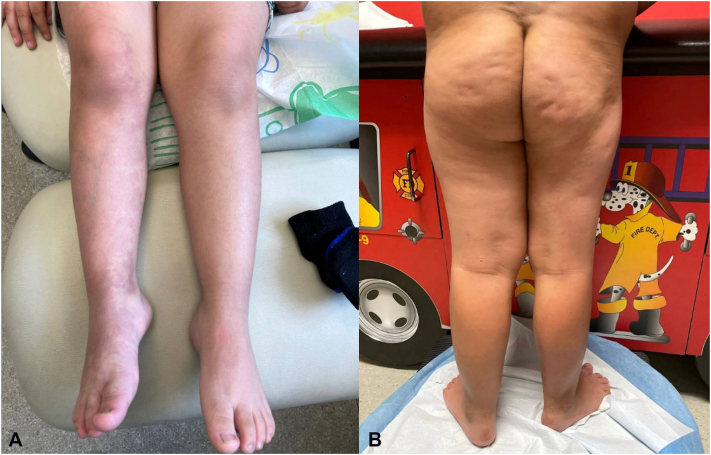
Hypoplastic lower extremity in PIK3CA-related altered growth spectrum. **(A)** Anterior view of the lower extremities at age 6 demonstrating relative hypoplasia of the right leg compared with the left, with associated capillary-venous malformations. **(B)** Posterior view demonstrating right-sided soft tissue hypoplasia and extensive capillary-venous malformations extending from the buttock to the foot. The left lower extremity demonstrates normal proportions.

Re-evaluation at age 6 demonstrated that the previously presumed left-sided hypertrophy represented normal limb proportions, while the right leg exhibited relative hypoplasia. Prior measurements at age 4 documented an anterior superior iliac spine-to-medial malleolus distance of 295 mm on the left versus 280 mm on the right, and thigh circumferences of 265 mm (L) vs 250 mm (R), confirming persistent underdevelopment of the right lower extremity. Vascular malformations remained stable on comparison with earlier imaging. Fig 1 illustrates the clinical asymmetry.

Although prior imaging described combined arteriovenous and capillary-venous malformations, representative magnetic resonance imaging performed at an outside institution demonstrated no significant deep vascular malformation of the muscle or soft tissue, with only subtle prominence of subcutaneous fat in the contralateral lower extremity.

### Interpretation

Given the presence of a right-sided *PIK3CA* variant, stable vascular malformations, and evolving right-sided underdevelopment, the findings were consistent with a PIK3CA-related undergrowth phenotype aligning with entities described within the PRAGS. Care was further complicated by fragmented follow-up across multiple institutions.

## Discussion

Activating *PIK3CA* variants drive aberrant growth through dysregulation of the PI3K-AKT-mTOR pathway, resulting in a spectrum of segmental vascular and growth abnormalities.^3^^,^^4^ While the PROS classification historically emphasized hypertrophic phenotypes,^1^^,^^3^ emerging literature, including the recent report by Ries et al, demonstrates that activating *PIK3CA* variants can also produce tissue underdevelopment, supporting the broader PRAGS framework.^2^

Our patient’s phenotype illustrates this complexity. Although initially characterized as left-sided hypertrophy, updated measurements and reevaluation confirmed that limb asymmetry resulted from right-sided hypoplasia. Similar hypoplastic presentations have been described in patients with mosaic *PIK3CA* variants, including hypoplastic toes, limbs, and soft tissues.^6^

Growth abnormalities associated with PIK3CA-related vascular malformations may occur ipsilateral or contralateral to the affected vascular territory, underscoring the importance of longitudinal assessment over reliance on initial laterality.^2^^,^^6^

These phenotypes may reflect early developmental timing of the variant, low variant allele fraction, vascular insufficiency from associated malformations,^7^ or competitive interactions between variant-bearing and wild-type cell populations during embryogenesis. Inconsistent access to specialty follow-up and interruptions related to multi-institutional care and insurance challenges may also influence clinical course.

The p.Met1043Ile variant identified in our patient has been documented in both overgrowth and undergrowth presentations,^8^ indicating that phenotypic expression depends more on spatial mosaicism and tissue involvement than on variant type alone.

Although alpelisib is effective for reducing progressive overgrowth in PROS,^9^ its role in established hypoplastic phenotypes remains unclear. Case series report minimal structural improvement of hypoplastic bone despite therapy,^6^ suggesting that once tissue underdevelopment is established, PIK3*a* inhibition has limited corrective potential. In our patient, limb hypoplasia persisted despite a prior trial of alpelisib.

For dermatologists evaluating vascular birthmarks with associated limb asymmetry, this case emphasizes several clinical pearls. First, limb asymmetry in the setting of segmental vascular malformations should prompt consideration of mosaic *PIK3CA* variants, even when the affected limb appears smaller rather than larger. Second, longitudinal measurements are critical, as initial assessments may misattribute asymmetry to overgrowth of the unaffected side. Third, genetic confirmation through lesional tissue sampling provides definitive diagnosis and informs prognosis and management decisions. Finally, multidisciplinary coordination between dermatology, genetics, and orthopedics is essential for comprehensive care of these complex patients.

This case highlights diagnostic challenges in PRAGS, particularly when early evaluations focus exclusively on overgrowth. Accurate characterization, supported by targeted sequencing of affected tissue, remains essential for diagnosis, prognosis, and therapeutic planning.

## Conflicts of interest

None disclosed.
